# Robustness evaluation of an inkjet‐printed epidermal ultra‐high‐frequency radio frequency identification tag

**DOI:** 10.1049/htl2.12002

**Published:** 2021-01-20

**Authors:** Dumtoochukwu Obiora Oyeka, John C. Batchelor

**Affiliations:** ^1^ Department of Electronic and Computer Engineering University of Nigeria Nsukka Enugu Nigeria; ^2^ School of Engineering and Digital Arts University of Kent Canterbury UK

## 1 INTRODUCTION

Radio frequency identification (RFID) is expected to have an important function in the growth of the Internet of Things in the 21st century. This is as a result of some unique features it has, such as very low power requirements as well as possibility of being used as passive and even active sensors. In recent years, RFID has found increased use on the human body [1, 2] often as sensors [3], in healthcare applications [4] or for tracking purposes [5]. This close interaction between the tag and the human body has been reported in [6].

Factors such are comfort and conformity are essential for body mounted tags, which, fortunately, are provided by inkjet‐printed tags on tattoo transfer paper [7], due to their very thin profile. This also means that the tags could be susceptible to wear and tear—leading to a cut in the feedline, loosening of the chip attachment point and also the effects of physiological factors such as sweating. Even though these are the factors that would not apply to less flexible tags, benefits of using inkjet printing to fabricate these tags far outweigh its limitations, and more so due to the tags’ temporary nature.

Considering the above, it is, therefore, necessary to assess the limits of body‐mounted tags to understand the limits of use. The tag robustness was tested based on performance during normal daily activities, after gym activities, after showering and the effects of sweat. Part of this work has been presented in [8].

## 2 EPIDERMAL ULTRA‐HIGH‐FREQUENCY RFID TAG DESIGN AND FABRICATION

**FIGURE 1:**
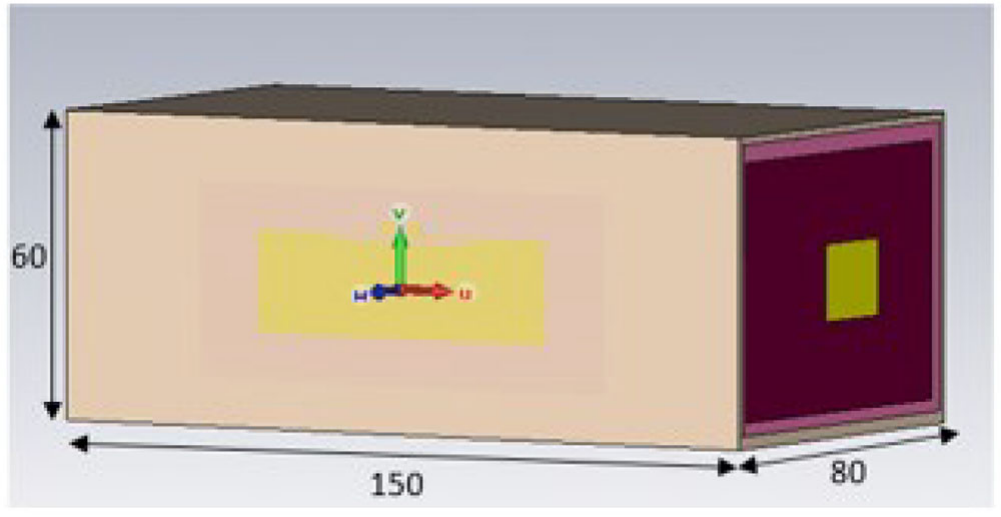
Four‐layer human phantom used to improve simulation accuracy (dimensions in mm)

The RFID tag was simulated on skin using Computer Simulation Technology™ Microwave studio. This software enables the designer to model and simulate the RFID over a frequency range and assess the quality of the impedance match to the application‐specific integrated circuit (ASIC) (or RFID transponder) used. The tag was modelled on a four‐layer phantom Figure 1), representing the skin, fatty layer, muscle and bone. Each layer was given electromagnetic properties as obtained from [9]. A conductivity of 2.73E+06 S/m was used for the antenna conductor. This represents two layers of conductive ink sintered for 20 min at 150 ^o^C.

**FIGURE 2:**
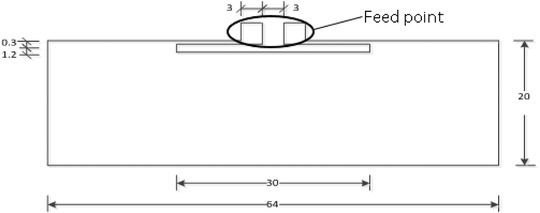
Designed slot transfer tattoo tag (dimensions in mm)

The tag design, shown in Figure 2 [9, 10], can be regarded as a slot antenna, with strong currents flow around the slot and with reduced current magnitude across the conductive body of the antenna. The narrow slot design results in acceptable performance when directly mounted on skin (with no shielding ground plane) when compared to alternative designs [11]. These features ensure that the tag would function in an electrically challenging environment, which the human body presents. The tag was designed to resonate at 866–868 MHz.

**FIGURE 3:**
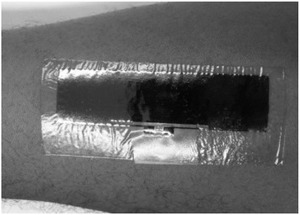
Inkjet‐printed tag transferred on arm

The fabrication was done with conductive silver nano‐particles ink [12, 13] inkjet printed onto transfer tattoo paper comprising a thin layer of polymer, which serves as a direct base for the deposited ink. Adhesive tape attached the ASIC to the printed tag. For attachment to the skin, a 5‐μm‐thick transparent adhesive film was applied to the printed tag. The tag was then mounted on the skin and the backing paper is made slightly damp to facilitate its removal, leaving the printed tag on the body. Figure 3 shows the final stage of this process.

## 3 EXPERIMENTAL SETUP

The tag was then tested under various conditions such as normal 8‐h working day as well as during exercise activities in a gym. For the full work day tests, the measurements were recorded every 30 min for the 8 h. Due to the temporary nature of this tag, a minimum of 8‐h functionality under non‐stressful conditions was chosen as a benchmark to determine satisfactory performance. The measurements for the robustness test for the exercise activities were taken after 20 min of various gym activities. The tag was placed on the torso of the volunteer in order to guarantee sufficient contact with sweat and also maximize the effect of body motion on the tag during the exercises. The performance of the tag when worn to sleep for 6 h was also assessed. All measurements were taken using Voyantic Tagformance kit [13]. This is a system that uses the Friis equation to extrapolate the read range by measuring the backscattered power from the tag and transmitted power that it uses to determine chip threshold power.

**FIGURE 4:**
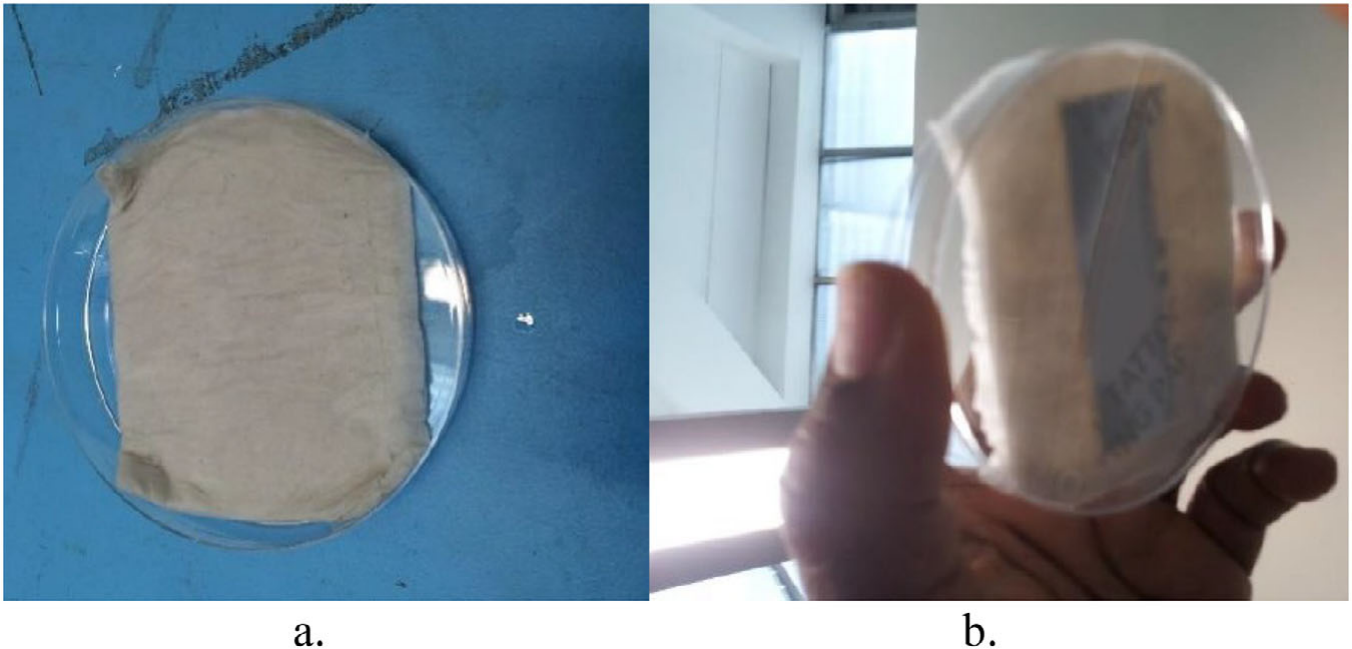
Test setup for effect of sweat on tag. (a) Top view. (b) Bottom view

It was also sought to determine if there is a specific effect of sweat on the deterioration of the tag or if the observed deterioration was simply due to wear and tear. Consequently, a more regulated experiment was set up in order to remove the effect of movements and, consequently, friction on the tag. This experiment would also help to establish if sweat had any effect on tag durability as against ordinary water (moisture). In order to achieve this, a saline solution was made to have a close similarity to sweat. The salt concentration in this solution was obtained from [14]. Of the two tags utilized for this experiment, one subjected to the saline solution, while the second tag was exposed to ordinary water. This was achieved by moistening two different cuts of tissue paper: one with the saline solution and the other with water. The tissue paper cuts were then placed on the tag samples. To ensure uniformity, 4 mL each of the saline solution and water were measured using a graduated cylinder before applying the tissue paper. The volume ensured that the tissue paper was not excessively soaked. The means of application of the tissue paper on the tag is shown in Figure 4(a) and (b).

## 4 EXPERIMENTAL RESULTS

**FIGURE 5:**
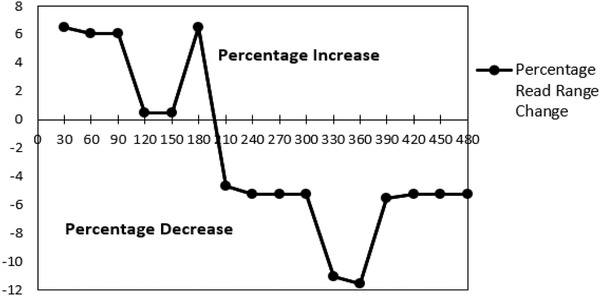
Tag read range measured over 8 h of normal work day

Figure 5 shows the read range measurement result of the tag after 8 h with 30‐min measurement intervals.

**FIGURE 6:**
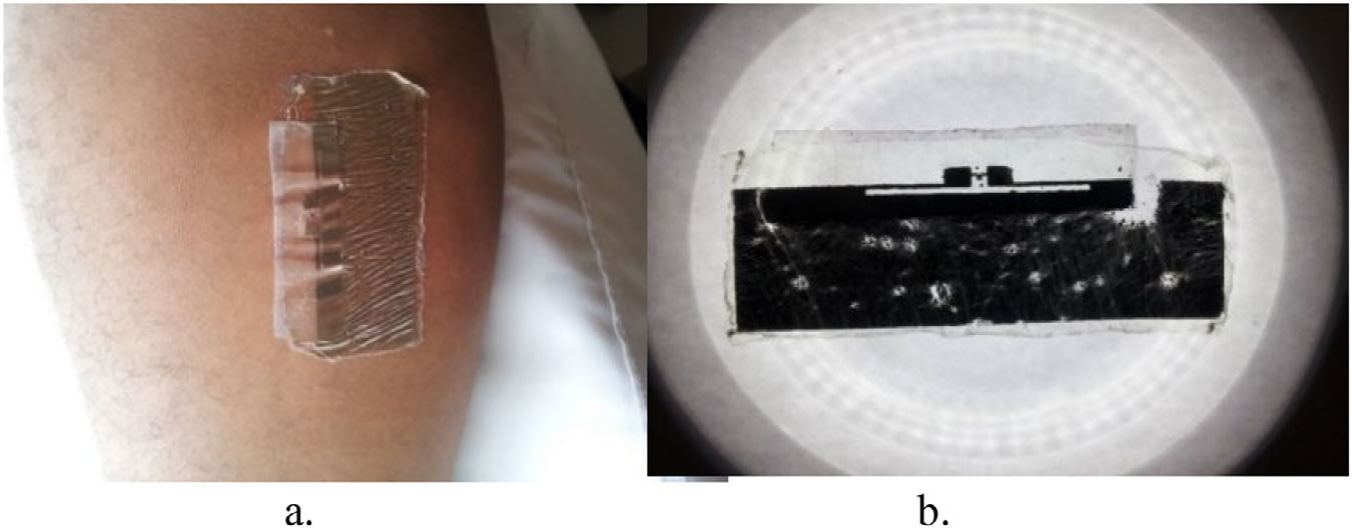
Tag robustness test. (a) After full day + sleep. (b) After full day + sleep on a light box

The first measured read range of the tag was 0.6 m. While there was no decrease in the measured read range during the first 180 min, subsequently, there are observed drops in the read range obtained from the tag. These drops in the read range could be attributed to wear and tear on the tag due to body movements by the user. This could lead to the weakening of the attachment of the chip to the tag as well as introduction of creases, which could lead to an increase in tag impedance. The effect of this is increased mismatch between the antenna and the chip leading to a decrease in the read range. It is necessary to note that the apparent increase in the read range noticed in the early stages of the measurement could be attributed to variations in the placement of the tag during the measurement as slight changes in the separation between the tag and the reader antenna would result to an apparent increase in the read range. The Voyantic Tagformance kit operates by measuring the backscattered power from the tag under test, while the transmitted power from the kit is used to set the threshold power of the chip in relation to frequency. With these values, the read range can then be deduced by the equipment utilising Friis equation because the associated losses from the cable and other sources have been removed prior to calculations during calibration. Tests indicate that a 1‐cm change in tag location after calibration would lead to about 20‐cm variation in the read range measured. Consequently, care was taken to avoid movements during the measurements. The tag was worn on the forearm to sleep but had suffered severe abrasion leading to the cutting of the feedline of the tag [see Figure 6(a) and (b)]. No reading was obtained from it afterwards. Hence, sleeping with the tag on offered the worst case of tag deterioration observed.

**FIGURE 7:**
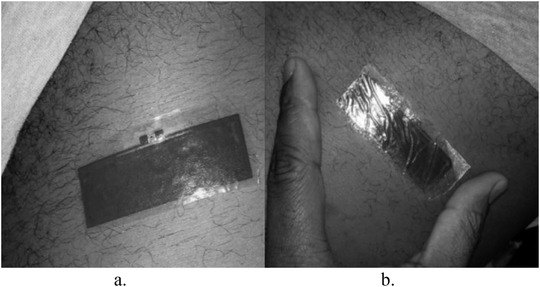
Tattoo tag attached to the torso for gym test. (a) Before. (b) After

The extended duration (8 h) of the tag's functionality is not indicative of an absolute test of robustness when considering its requirement for use in harsh environments such as during gym exercises. Consequently, a performance test was carried out during gym activities, which lasted for 20 min, which included 10 min on the treadmill and 10 min using the elliptical trainer. The before and after pictures of this experiment are shown in Figure 7(a) and (b), respectively.

**FIGURE 8:**
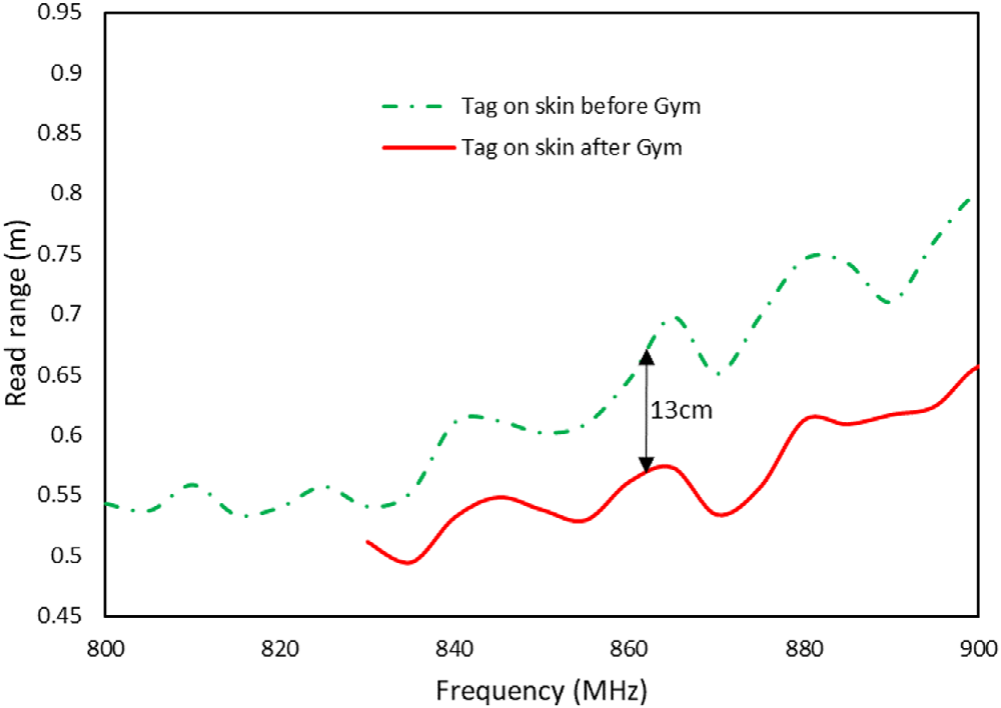
Tag read range measurement before and immediately after gym activities

Read range measurements afterwards showed a decrease in the read range when compared to the earlier measurements. Observation after the gym exercises shows the appearance of creases across the tag. However, because of the rigorous nature of the exercise and the associated movement of the torso, the creases were more pronounced than what was noticed in the earlier 8‐h test and also appeared in shorter time. As noted earlier, these creases have the effect of weakening the adhesion between the antenna and the chip as well as causing an increase in tag impedance. Even though the initial read range was measured to be about 70 cm, the measurement after exercising at the gym showed a reduction by about 13 cm, as seen in Figure 8. This decrease in the tag read range was not deemed to be adverse because the final read range was about 57 cm (approximately 19% decrease). For such a tag that operates in very close proximity with the human body and considering the challenges therein, a 50‐cm read range is considered satisfactory since the tag is to be used for short distance purposes.

**FIGURE 9:**
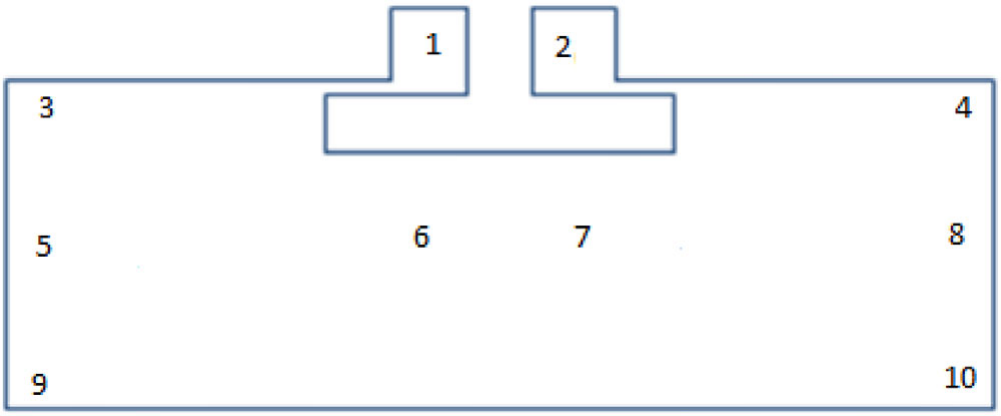
Resistance measurement points for effect of sweat on tag

Prior to assessing the effects of sweat, measuring points were defined on the tags according to Figure 9.

**FIGURE 10:**
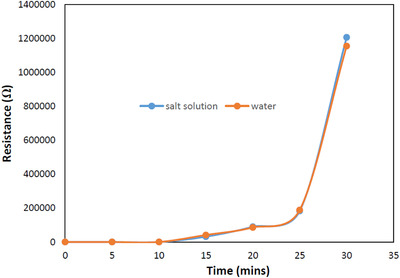
Resistance measurements of sample tags in water and salt solution

Point‐to‐point resistance measurements were carried out at these points before the start of the experiment and then at 5‐min intervals for 30 min using the order: 1–2, 1–3, 2–4, 3–9, 4–10, 3–10, 4–9, 5–6, 6–7 and 7–8. The averages of the resistances were taken for every measurement, and the results are plotted in Figure 10.

The results of the point‐to‐point resistance measurements indicate that no notable dissimilarity can be observed between the two tags. This observation would lead to the conclusion that the degradation of the tag during the gym activities had more to do with motion and friction than the effect of subjection to sweat. The first 15 min of the measurement where both tags were intact gave the most confidence in the results. This is because after this point, cracks were observed on both tags, which resulted to increase in resistance values. In addition, there was also folding of the paper underneath the polymer layer, where the conductive ink was deposited due to exposure to both liquids.

**FIGURE 11:**
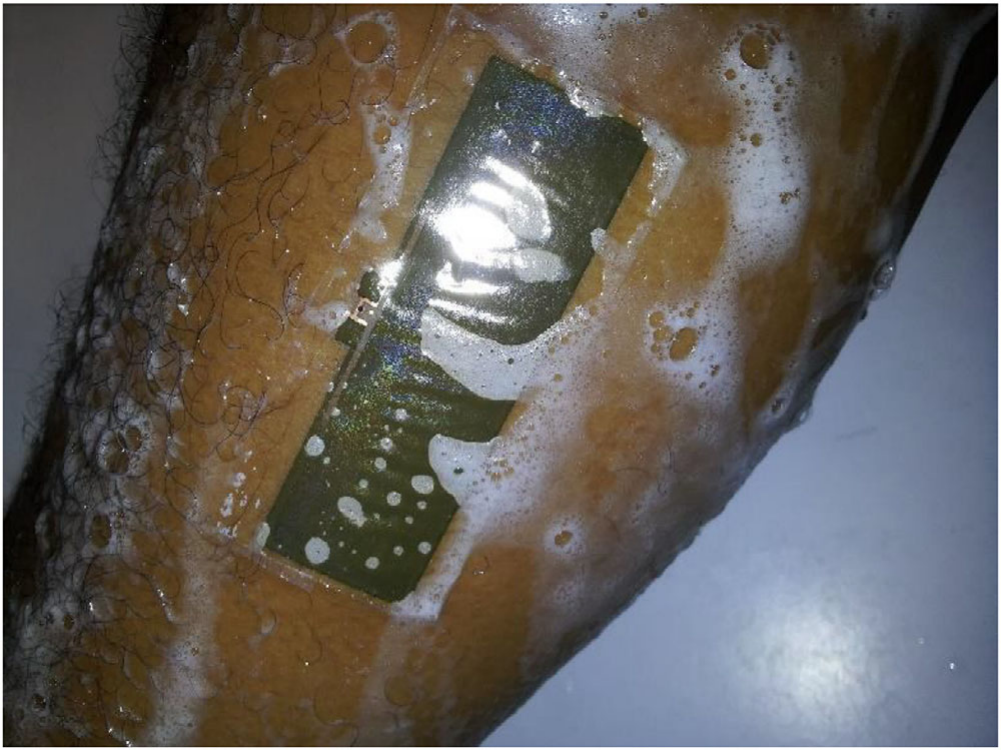
Transfer tattoo tag covered with soap during shower test

**FIGURE 12:**
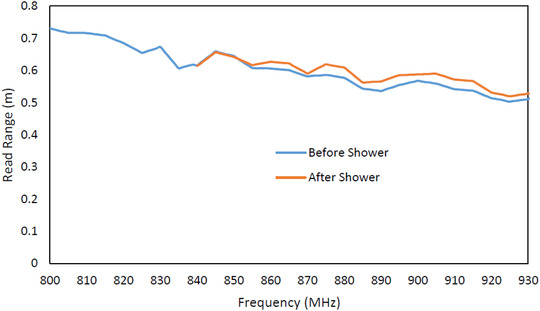
Measured read range of transfer tattoo tag before and after shower

In order to assess further the effect of exposure to liquid on the tag, it was worn during a shower for 10 min. This had the effect of exposing the transfer tattoo tag to water as well as bathing soap. Caution was taken to avoid exposing the tag to vigorous rubbing. Figure 11 shows the image for this experiment, while Figure 12 shows the measurement results.

**FIGURE 13:**
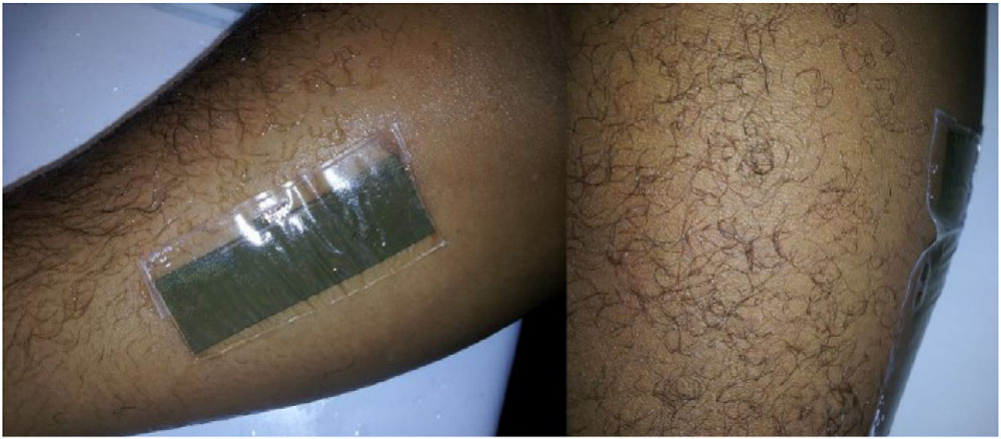
Transfer tattoo tag showing air gap after shower test

Figure 12 shows the tag read range results before and after shower. The result suggests that the tag performance remains relatively unaffected after it was exposed to water when it was mounted on skin. It can be seen from the graph that there is a slight improvement in the read range of the tag after shower. This improvement in the read range is because the adhesion between the tag and the skin became weakened, hence enabling the formation of an air gap between the two surfaces (see Figure 13). This separation between the tag and the skin, therefore, reduced the influence of the skin on the tag's performance, which explains the observed increase in the measured read range. It can, therefore, be said that while showering had no adverse effect on the tag itself, the adhesion between the tag and the body was affected.

## 5 CONCLUSION

In this work, the robustness tests were conducted to assess the durability of an inkjet‐printed epidermal RFID tag. These tests carried out over an 8‐h period. Read range measurements showed a non‐uniform step decrease in the tag read range, but tag operation was continuous throughout the duration of the test. However, the tag was badly damaged when worn overnight due to a cut on the feedline. The tag still functioned after gym exercise, though it showed signs of physical degradation. Further tests showed that the decrease in the tag read range was more as a result of wear and tear due to mechanical friction than being subjected to sweat, as DC resistance measurements showed no notable variation between exposing the tag to sweat (saline solution) and to water for a period of 30 min. Finally, exposing the tag to water and bathing soap during showering resulted in no decrease in the tag read range. These results have gone on to show that tags such as the one presented here have shown some promise for use on the human body. However, future body‐mounted inkjet‐printed tags would require more robustness in the conducting pattern while striving to maintain skin comfort.
